# Identification of a Candidate Gene for the Novel Cytoplasmic Male Sterility Derived from Inter-Subspecific Crosses in Rice (*Oryza sativa* L.)

**DOI:** 10.3390/genes12040590

**Published:** 2021-04-17

**Authors:** Zhuo Jin, Jeonghwan Seo, Backki Kim, Seung Young Lee, Hee-Jong Koh

**Affiliations:** 1 Department of Agriculture, Forestry and Bioresources, Plant Genomics and Breeding Institute, Research Institute for Agriculture and Life Sciences, Seoul National University, Seoul 08826, Korea; jinzhuo0116@gmail.com (Z.J.); rightseo@hotmail.com (J.S.); uptfamily@hanmail.net (B.K.); sngyngl9@gmail.com (S.Y.L.); 2 Department of Plant Bioscience, College of Natural Resources and Life Science, Pusan National University, Miryang 50463, Korea

**Keywords:** cytoplasmic male sterility, *orf312*, Tetep-CMS, mitochondrial genome, mitotype-specific sequences, restorer-of-fertility

## Abstract

Tetep-cytoplasmic male sterility (CMS) was developed through successive backcrosses between subspecies *indica* and *japonica* in rice (*Oryza sativa* L.), which showed abnormal anther dehiscence phenotypes. Whole genome sequencing and de novo assembly of the mitochondrial genome identified the chimeric gene *orf312*, which possesses a transmembrane domain and overlaps with two mitotype-specific sequences (MSSs) that are unique to the Tetep-CMS line. The encoded peptide of *orf312* was toxic to *Escherichia coli* and inhibited cell growth compared to the control under isopropyl-β-D-1-thiogalactopyranoside (IPTG) induction. The peptide of *orf312* contains COX11-interaction domains, which are thought to be a main functional domain for *WA352c* in the wild abortive (WA-CMS) line of rice. A QTL for *Rf-Tetep* (restorer-of-fertility gene(s) originating from Tetep) was identified on chromosome 10. In this region, several restorer genes, *Rf1a, Rf1b,* and *Rf4,* have previously been reported. Collectively, the interactions of *orf312*, a candidate gene for Tetep-CMS, and *Rf-Tetep*, a restorer QTL, confer male sterility and fertility restoration, respectively, which enables a hybrid rice breeding system. Further studies on *orf312* and isolation of *Rf-Tetep* will help to identify the underlying molecular mechanism of mitochondrial ORFs with the COX11-interaction domains.

## 1. Introduction

Cytoplasmic male sterility (CMS) has been found in more than 150 flowering plant species [1,2]. The CMS system is usually obtained through successive backcrosses of inter- or intra-subspecies [3]. The cytoplasms from male sterile CMS lines are identified when the nuclear genome is replaced by other cultivars. This maternally inherited trait is widely used in hybrid seed production as a female parent [4]. The fertility of F_1_ hybrids is restored by the nuclear *restorer-of-fertility* (*Rf*) genes, derived from the male parent [5]. The CMS/*Rf* system is also a good genetic resource for the study of mitochondria–nucleus interactions [3].

In rice, more than 60 types of CMS systems have been developed based on the origin of the cytoplasm [6]. Several CMS-related genes have been found in rice, *WA352c* and *WA314* from WA-CMS (wild abortive) [1,7]; *atp6-orf79* from BT-CMS (Chinsurah Boro Ⅱ) [8]; *atp6-orfH79* and *orf290* from HL-CMS (Honglian) [9,10]; *orf182* from D1-CMS (Dongxiang) [11]; *orf352* from RT102-CMS (RT102C) [12]; *orf113* from RT98-CMS (RT98C) [13]; *orf307* from CW-CMS (Chinese wild rice) [14]; *atp6-orfL79* from LD-CMS (Lead rice) [15]. The CMS genes found in rice are all chimeric genes segmented with essential genes or unknown resources [1,16,17]. Based on the chimeric structure, the CMS genes *WA352c*, *WA314*, *orf352*, and *orf307* partially share segments with a non-CMS gene, *orf314* from Nipponbare [1]. The N-terminal peptide of ORF307 has an identical structure to ORF314 [14]. The C-terminal peptides of WA314, WA352a, WA352c and ORF314 came from the same ancestral ORF284b, which includes COX11-interaction region 1 and 2 [1]. COX11-interaction regions are important domains for *WA352c* function [18]. The *atp6-orf79* structure is detected in three CMS types from BT, HL and LD [19]. The *orf79* was co-transcribed with *atp6*, and the CMS function of *orf79* was explained and confirmed in BT-CMS by knockout mutants using the mitoTALEN method [20]. Due to lack of studies on the chimeric structures of *orf113* and *orf182*, the sequence of *orf113* is from an unknown origin source and homologous sequences of *orf182* are only found in *Sorghum bicolor* mitochondrial genomes [11,13].

The pollen grain phenotype is one of the essential factors to determine the mechanism of CMS sterility. The D1-CMS is considered as a non-pollen-type, which results in a lack of pollen grains [11]. Abortion of WA-CMS pollen grains mainly occurs at the uninucleate stage and results in amorphous pollen grains [7]. HL-CMS pollen grains are spherical but unable to be stained with I_2_-KI [6]. On the other hand, the BT, LD, RT98, and CW-CMS pollens are stainable with I_2_-KI [8,13,21,22]. For the stainable pollens of BT and LD-CMS, the pollens show a lower intensity of staining compared to normal pollen grains [8,22]. However, the RT98 and CW-CMS only show differences in the pollen tube growth of the pistil [13,21].

CMS restoration occurs through interactions between mitochondria and the nucleus in the CMS/*Rf* system. *Rf* genes are known to be responsible for the suppression of CMS phenotypic expression at different molecular levels [17]. Several *Rf* genes have been identified in rice CMS systems: *Rf1a* and *Rf1b* for BT-CMS [8]; *Rf3* and *Rf4* for WA-CMS [23,24]; *Rf5* and *Rf6* for HL-CMS [25]; *Rf2* for LD-CMS [26]; *Rf102* for RT102-CMS [12]; *Rf17* for CW-CMS [21]. Among the known *Rf* genes, *Rf1a* (*Rf5*), *Rf1b*, *Rf2*, *Rf4*, *Rf6,* and *Rf17* have been cloned, and half of them have been revealed to be RNA-binding pentatricopeptide repeat proteins (PPRs) located on chromosome 10 [16]. Characterization of the *restorer-of-fertility-like* (*RFL*) sequences based on the presence of tandem arrays of 15 to 20 PPR motifs found two *RFL* genomic clusters on chromosome 10, within the region where *Rf1a*, *Rf1b* and *Rf4* are located [27].

We found male sterility in populations that were backcrossed between subspecies of rice, in which the CMS cytoplasm was derived from Tetep, an *indica* rice variety. Consequently, both CMS and restorer lines of *japonica* genetic background were developed through further backcrosses and selections. This study aimed to elucidate the genetic cause of CMS and to map the fertility restorer gene so that a new hybrid breeding system can be set up in *japonica* rice.

## 2. Materials and Methods

### 2.1. Plant Materials

Tetep, an *indica* Vietnamese cultivar, was used as the cytoplasm donor parent; CP-SLO, a tropical *japonica* was used for successive backcrossing with Tetep; Hopum, a high quality Korean temperate *japonica* [28], was used as a recurrent parent for developing the Hopum genetic background male sterile line; Hopum A, a Hopum genetic background cytoplasmic male sterile line; Hopum R, Tetep cytoplasm and Hopum genetic background restorer line selected from F_4_ of Hopum A/(Tetep/Hopum^4^). All materials were grown with conventional cultural practices in the test field and artificial crosses were conducted in a greenhouse at the experimental farm of Seoul National University in Suwon, Korea.

### 2.2. Pollen Assays

The pollen grains from mature spikelets were fixed in 70% ethanol and stained with 1% (*w*/*v*) I_2_-KI (potassium iodide) solution. The stained pollens were observed using a microscope (Olympus CX31, Olympus, Tokyo, Japan).

For in vitro pollen germination, pollen grains were collected on a glass slide in the pollen germination medium (20% sucrose, 10% PEG, 3 mM calcium nitrate, 40 mg/L boric acid) during the floret opening period. After incubation under dark conditions at room temperature for 1 h, the pollen grains were observed via a microscope.

Pollen tube growth was measured using the aniline blue staining method described by Li et al. [29] with some modifications. More than 20 pistils from Hopum and Hopum A, collected at 3 h after natural pollination, were fixed in Carnoy’s solution (30% chloroform, 10% acetic acid, and 57% ethanol) for 1 h. Samples were then washed 3 times with water, incubated in 1 mol/L KOH at 55 °C for 5 min, and then stained in 0.1% aniline blue solution at room temperature under dark conditions for 18 to 24 h. Fluorescent images were observed under UV light on a fluorescent microscope (Axiocam 506 color^®^, Zeiss, Göttingen, Germany). The images were edited using the Affinity photo program (Serif Europe Ltd., Nottingham, UK) with “recolour” options; the hue value was set to 180°.

### 2.3. Whole Genome Sequencing and Mitochondrial Genome de Novo Assembly

Total DNA for Hopum, Hopum A, and Tetep was extracted from the young leaves using the modified cetyltrimethylammonium bromide (CTAB) method [30]. The libraries for whole genome sequencing were prepared by using the NEXTflex TM Rapid DNA-Seq kit (Bio Scientific Corporation, Austin, TX, USA). Paired-end sequencing was performed using the Illumina HiSeq 2500 (Illumina, CA, USA) with 250 cycles at the National Instrumentation Center for Environmental Management (NICEM) of Seoul National University (NICEM, Seoul, Korea).

The de novo assembly of the Hopum A mitochondrial genome was analyzed at Phyzen (Phyzen Inc., Seongnam, Korea). Raw data were trimmed by CLC quality trim (version 4.21) with Q20 and the de novo assembly of the low coverage whole genome shotgun sequence (dnaLCW) method was used to assemble the mitochondrial genome. Gene annotation was predicted by GeSeq [31], BLAST search, and the Artemis annotation tool [32]. A graphical genome map was generated using the OGDRAW tool (https://chlorobox.mpimp-golm.mpg.de/OGDraw.html, accessed on 21 April 2020) [33]. The large repeats of the Tetep-CMS mitochondrial genome was analyzed by Vmatch program [34].

### 2.4. CMS Orf Prediction

The ORFs were identified using the Artemis software [32]; the threshold was set to 70, followed by the method previously described in WA-CMS [35], RT102-CMS [12], RT98-CMS [13], and BT-CMS [36]. The common ORFs which showed >99% identity to the Nipponbare mitochondrial genome (DDBJ accession DQ167400) via a local BLAST search with *e* values < 1 × 10^−5^ were discarded. The TMHMM server v2.0 (https://services.healthtech.dtu.dk/service.php?TMHMM-2.0, accessed on 14 November 2019) was used for transmembrane domain prediction of proteins encoded by the ORFs.

### 2.5. RT-PCR and Rapid Amplification of cDNA Ends (RACE)

Total RNA from spikelets, a day before anthesis, was extracted with RNAiso plus (Takara Korea Biomedical Inc., Seoul, Korea). Two micrograms of total RNA was treated with RNase-Free DNase Ⅰ (Takara Korea Biomedical Inc., Seoul, Korea), and reverse transcribed with ReverTra Ace™ qPCR RT Master Mix with gDNA Remover (Toyobo Co., Ltd., Osaka, Japan), the master mix in the kit contains random primers and oligo dT. RT-PCR was performed with Prime Taq DNA Polymerase (GeNet Bio, Daejeon, Korea) with total 20 µL volume containing: 1 µL of cDNA reaction, 1 pmoles ORF-specific primers, 1 mM dNTPs Mix, 1 U prime Taq DNA Polymerase, and 10× reaction buffer. The reaction was as follows: 94 °C for 5 min; 30 cycles of PCR (94 °C for 30 s, 55 °C for 30 s, 72 °C for 30 s); 72 °C for 5 min. The primers for *atp6* from Luo [7] were used as internal controls. The RT-PCR analyses were repeated three times with similar results and the primer sequences are listed in Table S1.

The SMARTer^®^ RACE 5′/3′ Kit (Takara Korea Biomedical Inc., Seoul, Korea) was used to identify the 5′ and 3′ mRNA termini of *orf312*. The 5′ and 3′ RACE-Ready cDNA was synthetized from the total RNA following the manufacturer’s protocol. For the random primers used for 5′ RACE-Ready cDNA synthesis, refer to Appendix D of the SMARTer^®^ RACE 5’/3’ Kit User Manual. The RACE was performed with two gene specific primers for *orf312*; the sequences of the primers are listed in Table S1. The products were cloned using the In-Fusion HD Cloning Kit. For each PCR band, 10 colonies were picked, and sequenced by Macrogen (Macrogen Korea, Seoul, Korea).

### 2.6. Mitotype-Specific Sequence (MSS) and Phylogenetic Analysis

MSSs were detected using the described method from Xie et al. [37]. The thirteen mitochondrial genome sequences were obtained from GenBank, EMBL and DDBJ: Nipponbare (GenBank: DQ167400), PA64S (GenBank: DQ167807), 9311 (GenBank: DQ167399), IR6888B (EMBL: JF281153), Hassawi (EMBL: JN861111), IR1112 (EMBL: JN861112), WA-CMS (EMBL: JF281154), BT-CMS (DDBJ: AP017385 and AP017386), CW-CMS (DDBJ: AP011076), LD-CMS (DDBJ: AP011077), RT98-CMS (DDBJ: AP012527), RT102-CMS (DDBJ: AP012528), and D1-CMS (GenBank: KY486275). Similar MSSs were detected for Nipponbare and PA64S, and similar MSSs were detected among Hassawi, IR1112, and IR6888b.

The unweighted pair group method with an arithmetic average (UPGMA) dendrogram based on MSSs was constructed using the Cavalli–Sforza and Edwards genetic distance [38]. The dendrogram was visualized in Molecular Evolutionary Genetics Analysis 7 (MEGA7) [39].

### 2.7. Plasmid Construction and Escherichia coli Growth Curve Assay

The *Escherichia coli* growth curve assay was performed with the methods described by previous research [40]. Full coding sequences of *orf312* were amplified from Hopum A by PCR with primer set (the primer sequences are listed in Table S1). The fragment was digested with *NcoI* and *XhoI* and ligated to the *pET28a* vector, constructing the plasmid *pET28a-orf312*. The *pET28a* and *pET28a-orf312* were transformed into the Rosetta2 (DE3) strain of *E. coli*. Expression in *E. coli* DE3 cells was induced with 1 mM isopropyl-β-D-1-thiogalactopyranoside (IPTG).

The transformants were incubated in LB medium at 37 °C overnight at 200 rpm. Fifty microliters of pre-culture was moved to 50 mL LB medium and incubated at 37 °C at 200 rpm. OD600 was monitored every hour, and when OD600 reached 0.3, the culture was separated into two equal subcultures. IPTG was added to one of them, at a final concentration of 1 mM. The cultures were incubated at 37 °C and 200 rpm. The cell density (OD600) was measured every 30 min by withdrawing aliquots at various time periods, and the OD600 was measured by UV/VIS spectrophotometer (Biotek Instruments, Inc., Winooski, VT, USA). The data were analyzed with one way ANOVA with Duncan’s multiple range test using SPSS software.

### 2.8. QTL-Seq Analysis

Two extreme bulk samples were constructed: the fertile pool (natural seed setting rate > 90%) and the sterile pool (natural seed setting rate < 10%) with 25 individuals for each bulk. The DNA was extracted using the modified CTAB method. DNA from each pool was evenly mixed based on the DNA concentration. Whole genome sequencing (Illumina-NovaSeq 6000) for the two bulked DNA samples was conducted at Macrogen (Macrogen Korea, Seoul, Korea). The QTL-seq pipeline (QTL-seq version 2.1.3) [41,42] was used for mapping the QTLs for *Rf* genes.

## 3. Results

### 3.1. Development of a Novel CMS and Rf System

Tetep-CMS was obtained through successive backcrosses between Tetep (*indica,* cytoplasm donor) and CP-SLO (tropical *japonica,* recurrent parent). Another round of successive backcrosses was performed between BC_7_F_1_ (Tetep/CP-SLO^8^) plants (cytoplasm donor) and Hopum (temperate *japonica,* recurrent parent) to BC_5_F_1_ and a CMS line with a Hopum genetic background was developed and designated to Hopum A (Figure S1a).

In order to develop restorer lines, a single plant from BC_3_F_1_ (Tetep/Hopum^4^) crosses which showed a >80% seed setting was crossed with Hopum A. In F_4_, one line that showed complete restoration of fertility against Hopum A was selected. The line was tested for restoration of fertility and named as Hopum R (Figure S1b). Additionally, a BC_3_F_2_ population (*n* = 270), harvested from the highly fertile BC_3_F_1_ plant, was planted in the test field for QTL-seq analysis to map the restorer gene(s) (Figure S1b).

**Figure 1:**
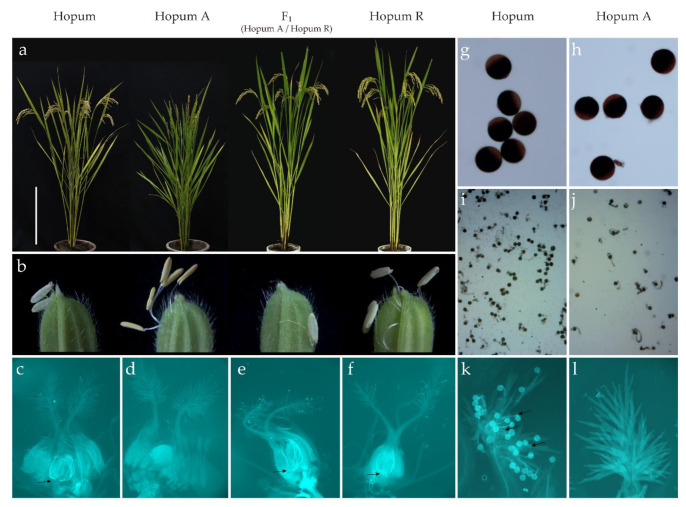
Phenotypes of plants. From left to right: Hopum, Hopum A, F_1_ (Hopum A/Hopum R), Hopum R, Hopum, and Hopum A. (**a**) Plants before harvest. The scale bar = 30 cm; (**b**) spikelet and anthers after spikelet closure; (**c**–**f**) pollen tubes can be observed in the ovule, the black arrow point to pollen tubes; (**g**,**h**) pollen staining with I_2_-KI; (**i**,**j**) in vitro pollen germination with germination medium; (**k**,**l**) pollen tube growth on the stigma, the black arrows point to pollen tubes in (**k**). F_1_: derived from Hopum A/Hopum R.

The plant height, culm length, and panicle length of Hopum A was significantly shorter than Hopum due to the effect of the cytoplasm. However, tiller number showed no significant differences (Table S2). After the maturing stage, the leaves of Hopum, F_1_ (derived from Hopum A/Hopum R), and Hopum R turned yellow, while the leaves of Hopum A were still green due to the lack of seeds set in the spikelets (Figure 1a). After anthesis, the pollen grains of Hopum, F_1_, and Hopum R were released and empty anthers were kept outside of the spikelet, while in Hopum A the anthers did not dehisce, even after the spikelets closed, and the anthers stayed yellow (Figure 1b). The growth of the pollen tube to the ovule was only observed in Hopum, F_1_, and Hopum R (Figure 1c–f) as no pollen was released from Hopum A and subsequently no pollen tube growth was observed on the pistil (Figure 1d). To compare the CMS and the maintainer lines, the pollen grains from both lines were stained with I_2_-KI solution (Figure 1g,h). During the in vitro pollen germination test, although fewer pollen grains were released from Hopum A (by forcefully shed to slide glasses), pollen grains from both Hopum and Hopum A germinated well on the germination medium (Figure 1i,j). However, on the stigma of Hopum A, due to anther indehiscence, no pollen grains came out from the anther and subsequently no pollen tube growth was observed in Hopum A (Figure 1k,l).

To validate whether the CMS was caused by abnormal anther dehiscence, a WA-CMS line Noksam A was used as the female parent, three panicles of Noksam A were artificially pollinated using Hopum A pollen and three panicles were bagged as the control. When Hopum A pollen grains were forcefully shed to Noksam A, seed setting on the panicles was observed while on the control no seed setting was observed, indicating that the pollen of Hopum A was viable. This suggests that cytoplasmic male sterility is caused by anther indehiscence in Tetep-CMS. Tetep-CMS was stably male-sterile under natural field and greenhouse conditions. However, more detailed environmental conditions affecting anther dehiscence remain to be studied.

### 3.2. Whole Genome Sequencing, Mitochondrial Genome de Novo Assembly, and MSS Analysis

**Figure 2:**
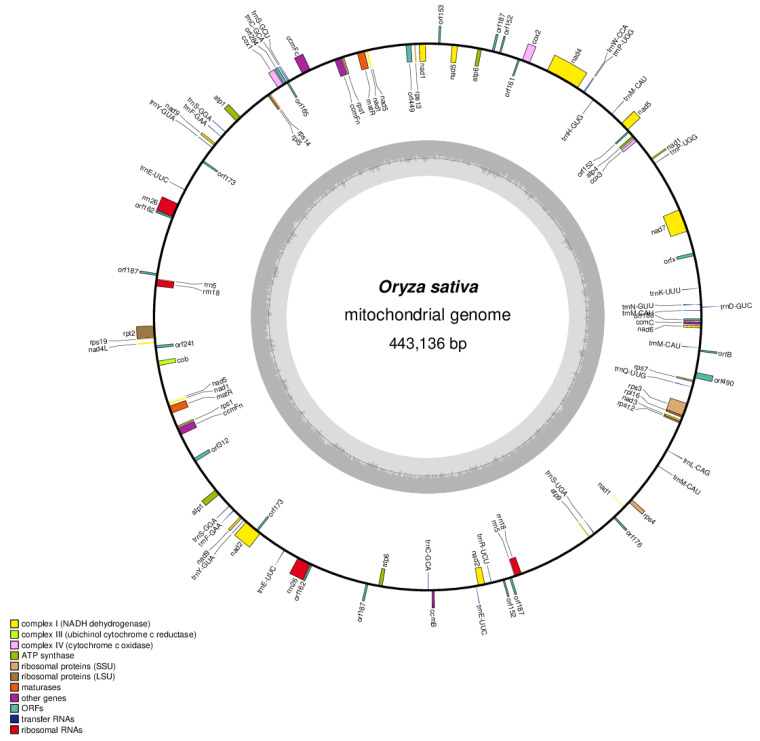
Physical map of mitochondrial genomes for Tetep-CMS line Hopum A. The outer circle represents the physical map. The genes on the inside (clockwise) or the outside (counter-clockwise) of the outer circle represent the transcription direction. Different colors indicate different genes as defined in the legend shown in left bottom of the figure. Complex Ⅰ, Ⅱ, Ⅲ, Ⅳ; ATP synthase; ribosomal proteins; maturases; other genes; ORFs; transfer RNAs and ribosomal RNAs.

To detect CMS-related genes for Tetep-CMS, whole genome sequencing was performed. We obtained three Illumina platform next generation sequencing (NGS) data sets for Hopum, Hopum A, and Tetep (Table S3). NGS data for Hopum A were used for mitochondrial genome de novo assembly. A total of 1,886,662 reads were filtered from Hopum A NGS data and assembled into a single circular molecule (443,136 bp) (GenBank accession number MW691120). A total of 32 protein coding genes, 17 tRNA genes, 3 rRNA genes, and 15 ORF genes were annotated (Figure 2, Table S4). Seven large repeats (>1 kb) were found in the Tetep-CMS mitochondrial genome, ranged from 2989 to 56,729 bp (Table S5). Paired end (PE) reads from Hopum, Hopum A, and Tetep were used to validate the draft mitochondrial genome. The complete mitochondrial genome was validated by the connectivity and consistency of the mapped reads. The structural variation was observed based on the depths plot. Unlike Hopum A and Tetep, which completely covered the draft mitochondrial genome, Hopum PE reads were not fully covered, with several gap regions (Figure S2).

The plant mitochondrial genome size varies within species [43]. Thirteen sequenced mitochondrial genomes in rice, including *japonica*, *indica,* and *O. rufipogon* varied in size ranging from 401,567 to 637,692 bp. Characterization of the mitotype can help to understand the structural variation and detection of CMS related genes [44]. Usually, CMS related genes are located in a unique MSS or overlap with some MSSs, for example, *orf79* (BT-CMS) and *orf182* (D1-CMS) were found within a unique MSS, and *WA352c* (WA-CMS) overlaps with two MSSs [11,37].

**Figure 3:**
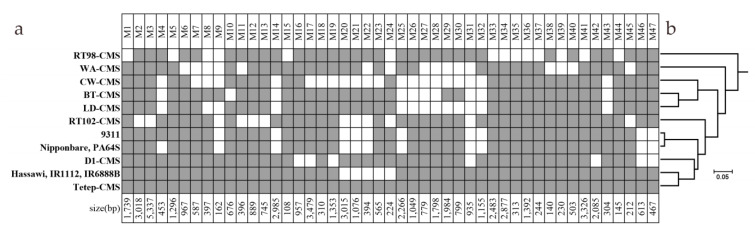
Distribution of MSSs specific to Tetep-CMS among the rice mitochondrial genomes. (**a**) Rice mitochondrial genomes with and without MSSs are represented by grey and white boxes, respectively. (**b**) UPGMA dendrogram based on 47 MSSs.

MSS analysis was performed by comparing the Tetep-CMS mitochondrial genome to 13 sequenced mitochondrial genomes from databases, including seven genomes of CMS lines and six genomes of fertile lines. The Tetep-CMS sequence was used as a query for the BLAST search. A total of 47 MSSs were detected, ranging from 108 to 5337 bp (Figure 3a). Five MSSs had duplicate copies in Tetep-CMS (M7, M12, M13, M14, and M15) (Table S6). There was no unique MSS identified in Tetep-CMS. Based on the phylogenetic tree, the Tetep-CMS mitochondrial genome shows high similarity to Hassawi, IR1112, and IR6888b mitochondrial genomes. Compared to Tetep-CMS mitochondrial genome, the mitochondrial genomes of Hassawi, IR1112, and IR6888b are lack five MSSs (M20 to M24) (Figure 3a,b).

### 3.3. ORF Identification and Validation with RT-PCR and RACE

**Figure 4:**
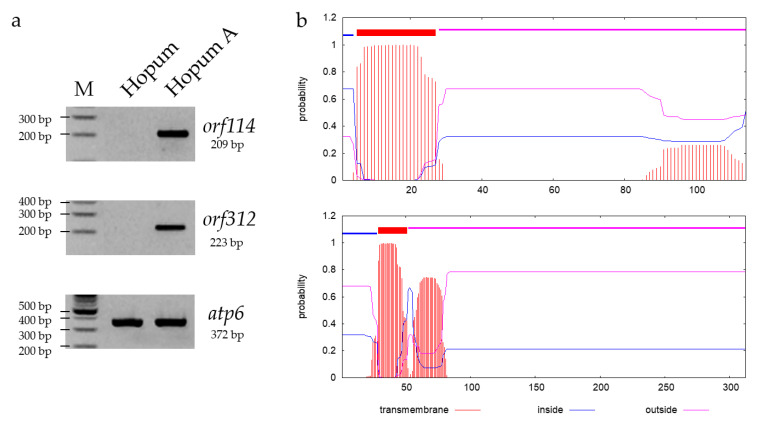
Candidate genes validation and transmembrane domains. (**a**) The RT-PCR results of *orf114* and *orf312*; *apt6* was used as the control; (**b**) transmembrane domain prediction results of ORF114 (up) and ORF312 (down). Protein sequences were analyzed using TMHMM server v.2.0 software. The output shows the location and probability associated with the predicted transmembrane domain.

Two ORFs, which contain transmembrane domains (Figure 4b), *orf114* and *orf312,* were specifically amplified in Hopum A by using specific primers which partially amplify the 209bp (34 to 242) from orf114, and 223bp (427 to 649) from orf312 gene segments. Therefore, these are considered as candidate genes for Tetep-CMS (Figure 4a). The BLAST search proved that *orf114* exists with 100% similarity in both *indica* and *rufipogon* mitochondrial genomes (fertile mitochondrial genome: Hassawi, IR1112, IR6888b, and 9311; CMS mitochondrial genome: RT102, D1, and WA-CMS). The *orf312* had no completely identical match in *indica*, *japonica,* or *O. rufipogon* mitochondrial genomes (Table S7). The location of the new identified chimeric gene *orf312* is 259,955–260,893 (reverse gene) in the Tetep-CMS mitochondrial genome, which overlaps with two MSSs: M23 and M24.

**Figure 5:**
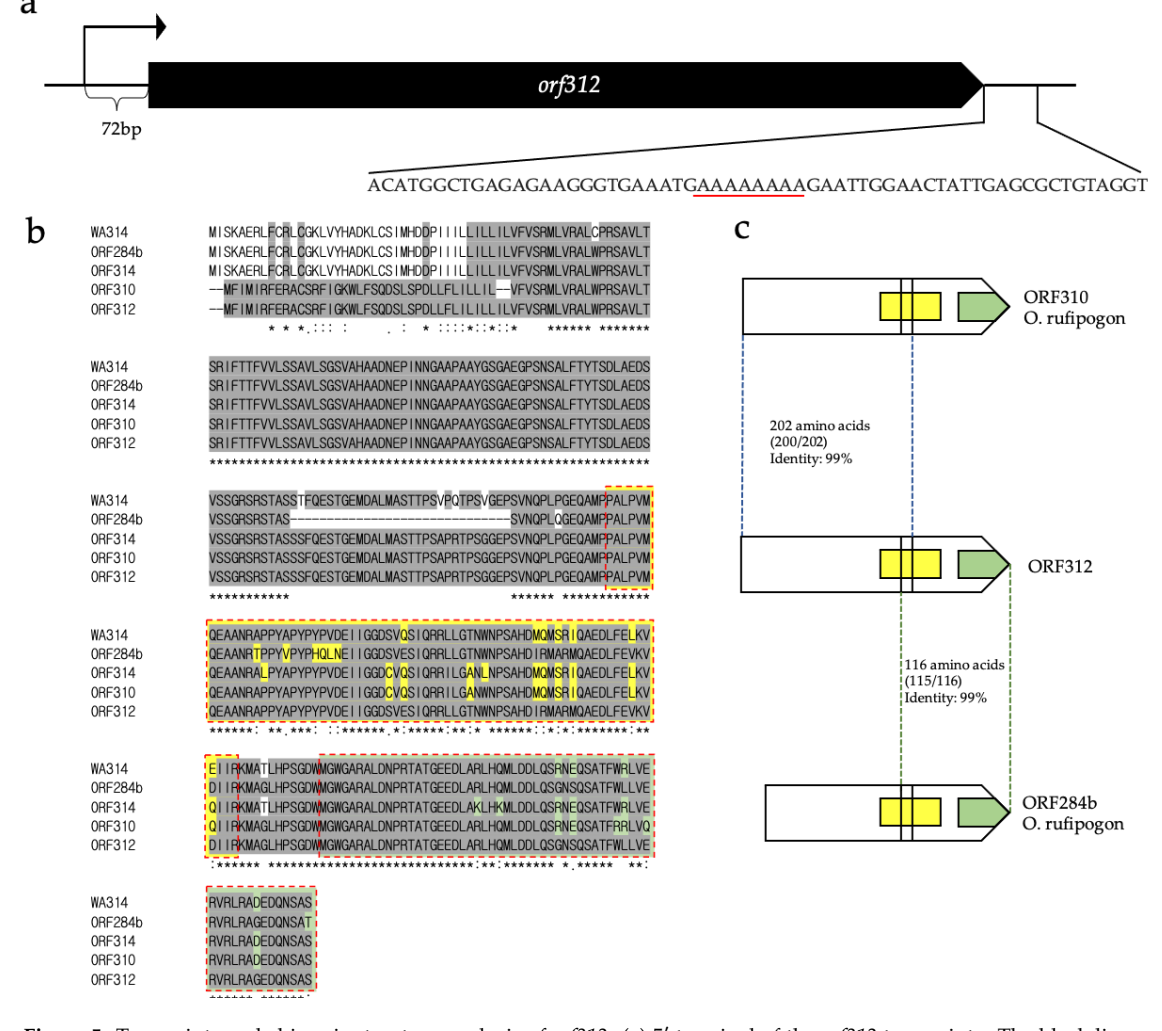
Transcripts and chimeric structure analysis of *orf312*. (**a**) 5′ terminal of the *orf312* transcripts. The black lines indicate *orf312* up and downstream sequences, and the black box indicates the coding sequence for *orf312*. The 5′ terminal of the *orf312* transcript is marked with arrows and the poly (A) sequence from downstream sequences is marked by red line. (**b**) Multiple protein sequence alignment with similar structures. The grey color shows completely matching sequences. The yellow and green boxes indicate COX11-interaction region 1 and 2, respectively; (**c**) chimeric structures of ORF312 protein. The N terminal 202 amino acids (1–202) show 99% similarity to ORF310. ORF310 has two amino acid deletions compared to ORF312, and the C terminal 116 amino acids (197–312) is 99% identical to ORF284b, only the last amino acid is different in ORF284b. The numbers in brackets show the number of identical amino acids/the number of total amino acids, and the yellow and green boxes represent COX11-interaction regions 1 and 2, respectively.

RACE was performed to identify the 5′ and 3′ ends of the *orf312* transcript. The 5′ end was located 72 bp upstream of the initial codon of *orf312* (Figure 5a) and the results of 3′ RACE showed an end which was located 26 bp downstream of the *orf312* stop codon. Usually the mitochondrial transcripts are missing poly (A), or the poly (A) was attached to transcripts for degradation [45]. We then checked the sequences downstream of *orf312* and we found the sequence of “AAAAAAAA” 26 bp downstream from the *orf312* stop codon (Figure 5a). The oligo dT may bind to this position to synthesize the first strand cDNA for 3′ RACE, that is why we identified the end position to “AAAAAAAA” sequence (Figure 5a). Although we did not identify the 3′ termini of the *orf312* transcript, the full coding sequence of *orf312* was validated by RACE. Based on the protein BLAST, four recombinants with >85% identities, proteins WA314, ORF314, ORF310 and ORF284b, were found in *O. rufipogon* (GenBank: KX255851, KX255855, KX255856, KX255857). Multiple protein sequence alignments showed that the N-terminal of ORF312 (1 to 202 amino acids) was identical to ORF310 except for two amino acid insertions, and the C-terminal of ORF312 (197 to 312 amino acids) was identical to that of ORF284b except for one amino acid change (Figure 5b, c). In addition, ORF312 contained chimeric COX11-interaction region 1 which consists of ORF310 and ORF284b, and the COX11-interaction region 2 was exactly same as that of ORF284b except for a single amino acid change.

### 3.4. ORF312 Expression in Escherichia coli

**Figure 6:**
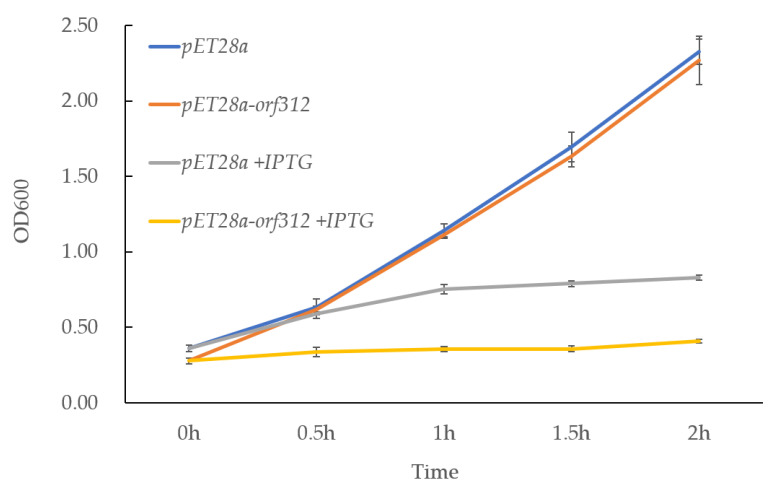
ORF312 expression in *E. coli:* cell growth in culture with or without IPTG induction. Cell density was determined at various time points for OD600. Cell were cultured at 37 °C. Data are presented as mean ± SD in triplicate.

Several coding peptides of CMS-related genes are cytotoxic and the encoded peptides inhibit the cell growth of *E. coli* [8,11,40]. During the induction experiment in liquid LB medium, the growth rate of the induced *pET28a-orf312* transformant was significantly repressed compared to the control. We tested cell growth by monitoring the OD600 every 30 min. Two transformants, *pET28a* and *pET28a-orf312*, showed similar growth rates without IPTG in liquid media. When IPTG was added, the growth of both transformants was reduced. In particular, growth of the *pET28a-orf312* transformant was significantly inhibited compared to the empty vector *pET28a* (Figure 6). These observations indicate that *orf312* encodes a cytotoxic peptide. When ORF312 was expressed in *E. coli*, the growth rate of *E. coli* was significantly reduced in comparison with the control.

### 3.5. QTL-Seq Analysis for the Restorer-of-Fertility (Rf) Gene

**Figure 7:**
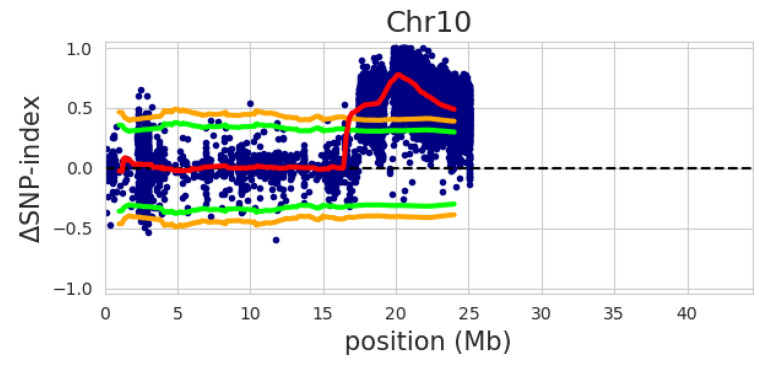
The deltaSNP-index plot of chromosome 10 with statistical confidence intervals under the null hypothesis of no QTLs (green, *p* < 0.05; orange, *p* < 0.01). The plot was generated using MH63RS2 as the reference genome with NGS data of Tetep as the parent, and fertile-bulk as bulk1, and sterile-bulk as bulk2.

Two pools of bulked DNA, from extreme fertile and sterile plants in a segregating BC_3_F_2_ population (Figure S1b), were sequenced using the Illumina platform with a minimum of 50x depths. The genetic backgrounds of the two pools were similar to the Hopum genetic background. To gain sufficient single nucleotide polymorphisms (SNPs), the NGS data of Tetep were used as the parent, the fertile-bulk as bulk1, and the sterile-bulk as bulk2. The Minghui63 sequence (MH63RS2) downloaded from the Rice Information GateWay (RIGW, https://rice.hzau.edu.cn/rice_rs3/, accessed on 10 December 2019) was used as the reference sequence. A QTL region was identified on chromosome 10 (Figure 7). We obtained SNP, insertion, and deletion (InDels) information on the region (from 17 Mb to 25.1 Mb in MH63RS2). *Rf* gene fine mapping is currently being conducted based on the SNPs and InDels for ultimate identification.

## 4. Discussion

This is the first report on mitochondrial gene *orf312,* which is associated with anther indehiscence and subsequent CMS in rice. Previously reported CMS related genes all cause male sterility, affecting pollen development [16]. In one CMS type in rice, the cytoplasm of which originates from Tadukan, the CMS is caused by anther indehiscence [46]. To date, there is no molecular study for CMS which caused by anther indehiscence. It has only been studied in pigeonpea (*Cajanus cajan* L. Millsp.), a mitochondrial gene (*orf147)* from the A4 cytoplasm reported to cause CMS by aberrant anther dehiscence; cytotoxicity and aberrant programmed cell death (PCD) could be important for the CMS mechanism in the A4 cytoplasm [47]. The peptide of *orf312* also showed cytotoxicity to *E. coli* and, as with *orf147*, it may have a relationship with CMS phenotypes in Tetep-CMS.

The peptide of *orf312* contains COX11-interaction domains. In previous studies, several CMS-associated genes, such as *WA314* and *WA352c* in WA-CMS and *orf352* in RT102-CMS, all contain COX11-interaction regions [1]. After I_2_-KI staining, RT102-CMS pollen grains appeared with unstainable shrunken and dark-stained spherical pollens, while only unstainable shrunken pollens were observed in WA-CMS [7,12]. In WA-CMS, the function of COX11 was suppressed by WA352c and resulted in premature PCD in tapetal cells at the unicellular stage, resulting in pollen abortion [7]. The COX11 protein may play a key role in anther wall development and degradation. The pollen grain phenotypes of WA, RT102, and Tetep-CMS may be all related to abnormal anther wall degradation. The CMS-associated genes, *WA314, WA352c, orf312*, and *orf352*, may interact with COX11 in different pollen development stages, resulting in varied phenotypes of pollen grains. The cytotoxicity and COX11-interactions could be important for the CMS mechanism in Tetep-CMS.

The upstream and downstream sequences of *orf312* were identical to the upstream and downstream sequences of *orf310*. In previous research, due to two amino acid differences (6 bp in nucleotides), the PCR bands from *orf312* and *orf310* have identical gel profiles, which explains why the *orf312* was not previously detected [1]. There may be more recombinants of *orf312*-like structures that have not yet been identified in rice germplasm. Whole genome sequencing data may help us to explore new recombinants in the future and give clear insights into mitochondrial recombination events. Furthermore, the functional study of new recombinants will provide us with an insight into how COX11-interaction domains are involved in CMS phenotypes.

We identified a QTL region in chromosome 10 for *restorer-of-fertility* against Hopum A. *Rf1a*, *Rf1b*, *Rf4*, and several PPR-coding *RFL* genes are located in this region. The rapid evolution of *RFL* genes plays an important role in the rapid evolution of mitochondrial genomes. The *RFL* genes suppress the abnormal transcripts from mitochondria to avoid nuclear–mitochondrial incompatibility such as CMS [27]. For example, *Rf1a*, *Rf1b* and *Rf4* function to decrease the levels of CMS-associated mRNA, *atp6-orf79* mRNA in BT-CMS and *WA352c* mRNA in WA-CMS, respectively. Currently, we are conducting fine mapping of the *Rf* gene, and the PPR genes in our QTL region are strong candidates for Tetep-CMS.

Heterosis of F_1_ hybrids in inter-subspecies is much higher than between intra-subspecies crosses. Additionally, heterosis of F_1_ hybrids between *indica* varieties is generally larger than the heterosis between *japonica* varieties [48,49]. This limits the usage of *japonica* hybrid varieties. There is a fundamental need to exploit higher heterosis in *japonica* rice through enhancing available hybrid seed production systems [50]. To apply Hopum A to inter-subspecific hybrid rice breeding, a widely compatible Hopum with neutral hybrid sterility genes such as *S24* and *S5* [51] was used as a maintainer line for breeding widely compatible Hopum A. It is expected that the widely compatible CMS lines can be applied to inter-subspecific hybrid rice breeding in the near future.

## 5. Conclusions

A novel CMS and *Rf* system was developed through the introduction of a sterile cytoplasm and restorer QTL of an *indica* variety, Tetep, into the *japonica* variety, Hopum, in rice. A chimeric gene, *orf312*, in the mitochondrial genome was a strong candidate gene for Tetep-CMS, which causes anther indehiscence and subsequent male sterility. The restorer QTL against the Tetep-CMS was mapped to chromosome 10.

## Data Availability

Tetep-CMS mitochondrial genome sequence obtained in this study is available in GenBank with accession number MW691120. All the Next Generation Sequencing raw data are available in the NCBI Short Read Archive (SRA) databases under the following BioProject accession numbers: Hopum (PRJNA705813), Hopum A (PRJNA705829), Tetep (PRJNA705822), Fertile-bulk (PRJNA705830), Sterile-bulk (PRJNA705841).

## Supplementary Materials

The following are available online at https://www.mdpi.com/article/10.3390/genes12040590/s1, Figure S1: Pedigree of Hopum A (a), Hopum R and QTL-seq analysis population (b), Figure S2: Read depth and coverage for Tetep-CMS mitochondrial genome, Figure S3: The deltaSNP-index plot for QTL-seq analysis, Table S1: Primers used in this study, Table S2: Agronomical traits for Hopum and Hopum A, Table S3: Summary of whole genome sequencing statistics, Table S4: Gene annotation in Tetep-CMS mitochondrial genome, Table S5: Large repeats in Tetep-CMS mitochondrial genome, Table S6: Location of MSSs in the Tetep-CMS mitochondrial genome, Table S7: BLAST hit table for *orf114* and *orf312*.

## Abbreviations

CMS	cytoplasmic male sterility
IPTG	isopropyl-β-D-1-thiogalactopyranoside
ORF	open reading frame
WA-CMS	Wild abortive CMS
BT-CMS	Chinsurah Boro Ⅱ CMS
HL-CMS	Honglian CMS
D1-CMS	Dongxiang wild rice CMS
CW-CMS	Chinese wild rice CMS
LD-CMS	Lead rice CMS
Rf	restorer-of-fertility
PPR	pentatricopeptide repeat proteins
RFL	restorer-of-fertility like
CTAB	cetyltrimethylammonium bromide
WGS	whole genome sequencing
dnaLCW	de novo assembly of low coverage WGS
NGS	next generation sequencing
PE	paired end
MSS	mitotype-specific sequence
PCD	programmed cell death
