# Supplementary material for: Identification of a Candidate Gene for the Novel Cytoplasmic Male Sterility Derived from Inter-Subspecific Crosses in Rice (Oryza sativa L.)
Source: Genes (Basel). 2021 Apr 17;12(4):590. doi: 10.3390/genes12040590 (PMC8073397; doi:10.3390/genes12040590)
Supplement: Supplementary file 1 [file genes-12-00590-s001.zip › Supplementary_Material_Figures_S1-S3(revised version).pptx]

## Slide 1
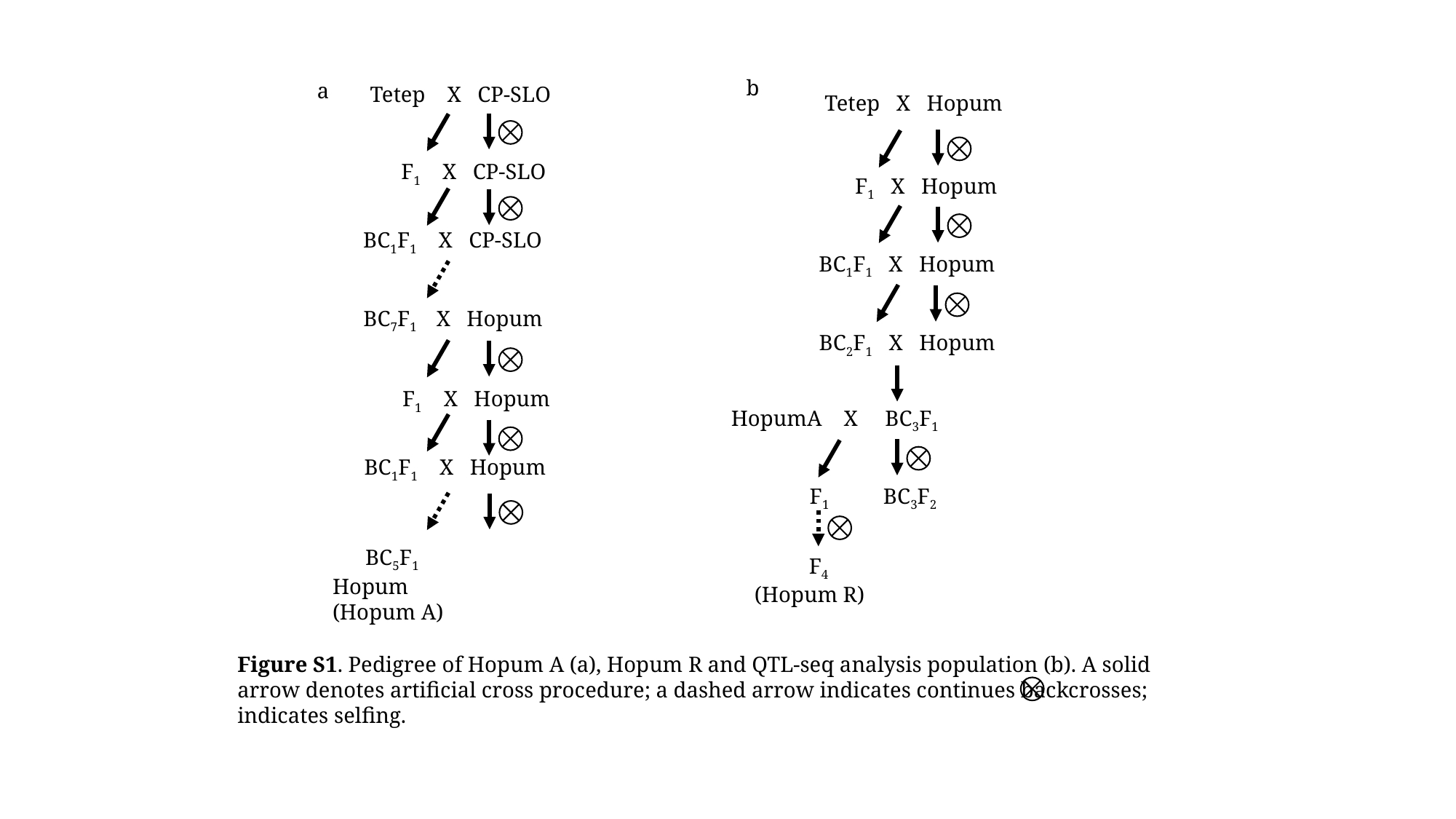

b
a
Tetep X CP-SLO
Tetep X Hopum
F1 X CP-SLO
F1 X Hopum
BC1F1 X CP-SLO
BC1F1 X Hopum
BC7F1 X Hopum
BC2F1 X Hopum
F1 X Hopum
HopumA X BC3F1
BC1F1 X Hopum
F1
BC3F2
 BC5F1 Hopum
(Hopum A)
 F4
(Hopum R)
Figure S1. Pedigree of Hopum A (a), Hopum R and QTL-seq analysis population (b). A solid arrow denotes artificial cross procedure; a dashed arrow indicates continues backcrosses; indicates selfing.

## Slide 2
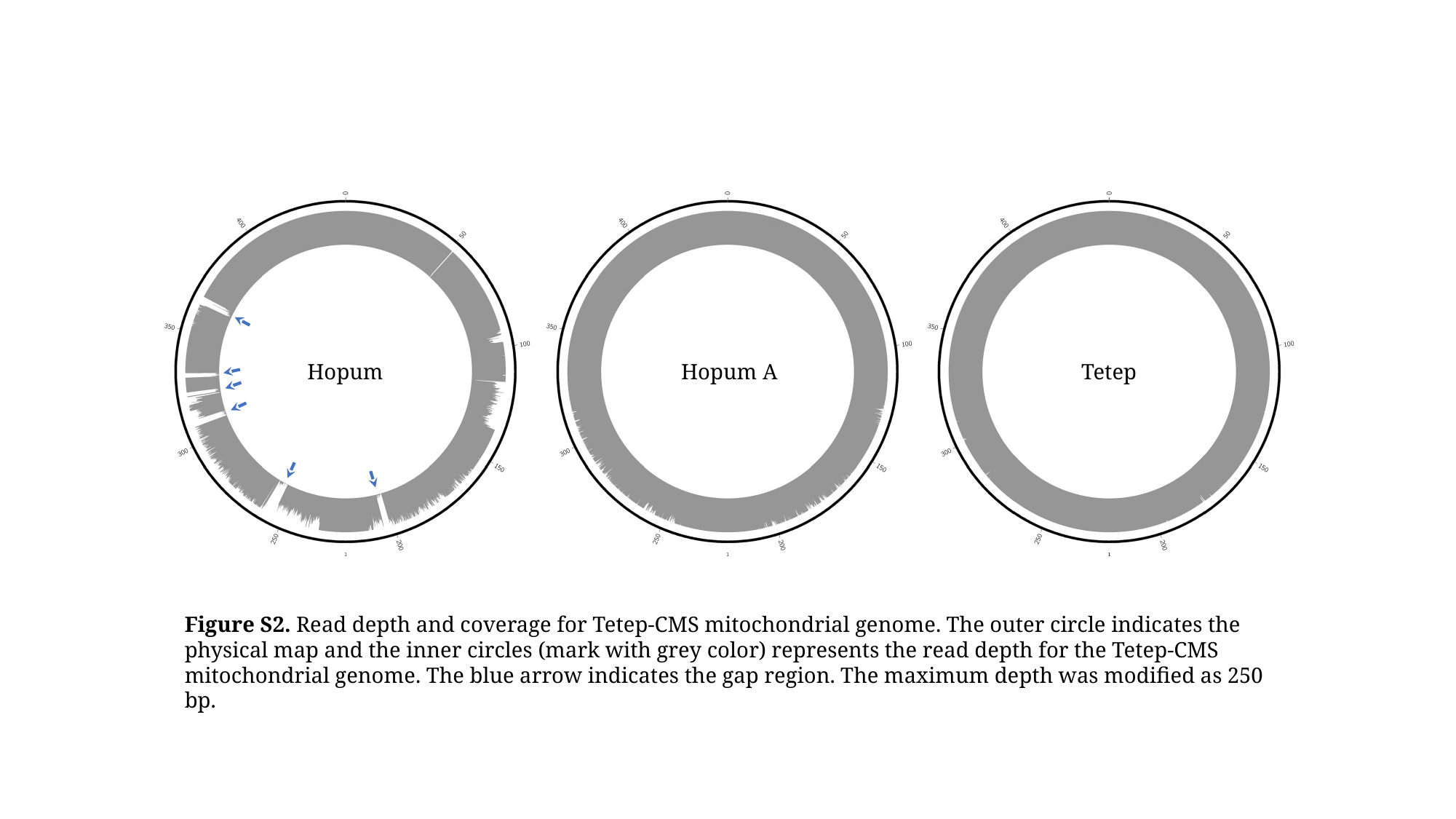

Hopum
Tetep
Hopum A
Figure S2. Read depth and coverage for Tetep-CMS mitochondrial genome. The outer circle indicates the physical map and the inner circles (mark with grey color) represents the read depth for the Tetep-CMS mitochondrial genome. The blue arrow indicates the gap region. The maximum depth was modified as 250 bp.

## Slide 3
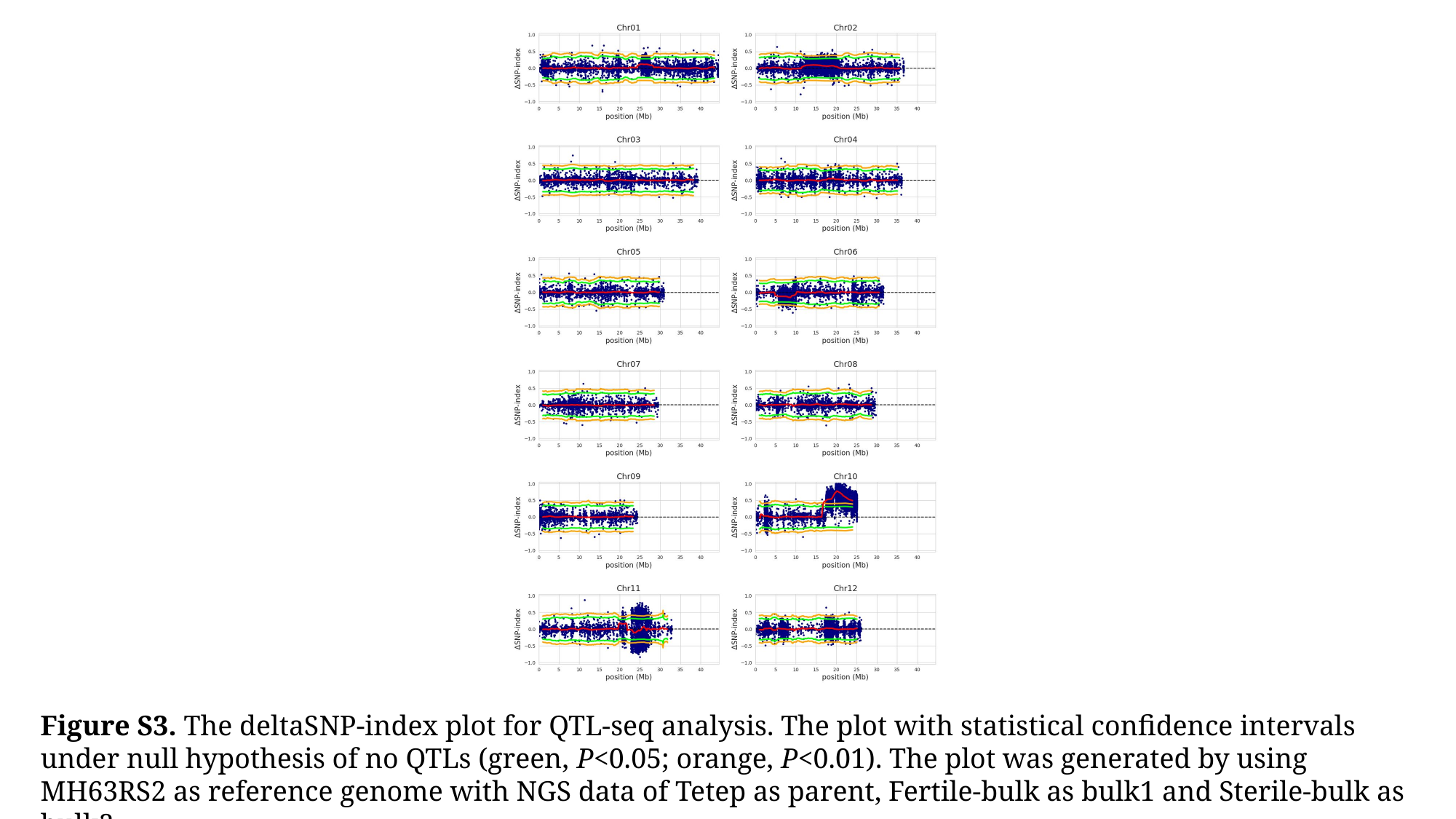

Figure S3. The deltaSNP-index plot for QTL-seq analysis. The plot with statistical confidence intervals under null hypothesis of no QTLs (green, P<0.05; orange, P<0.01). The plot was generated by using MH63RS2 as reference genome with NGS data of Tetep as parent, Fertile-bulk as bulk1 and Sterile-bulk as bulk2.
